# Pasteur Institute of Iran- An Evaluation Model

**DOI:** 10.6091/ibj.12472.2014

**Published:** 2014-07

**Authors:** Masoumeh Dejman, Elham Habibi, Monir Baradarn Eftekhari, Katayoun Falahat, Hossein Malekafzali

**Affiliations:** 1 *Social Determinants Health Research Center, University for Welfare and Rehabilitation Sciences, Tehran, Iran;*; 2 *Undersecretary for Research and Technology, Ministry of Health and Medical Education, Tehran, Iran; *; 3 *Dept. of Epidemiology and Biostatistics, School of Public Health, Tehran University of Medical Sciences, Tehran, Iran*

**Keywords:** Pasteur Institute, Iran, Indicator, Evaluation

## Abstract

**Background: **Pasteur Institute of Iran was established in 1919 with the aim to produce vaccines and prevent communicable diseases in Iran. Over time, their activities extended into areas of research, education and services. Naturally, such a vast development begs establishment of a comprehensive management and monitoring system. With this outlook, the present study was carried out with the aim to design a performance assessment model for Pasteur Institute of Iran that, in addition to determining evaluation indicators, it could prepare the necessary grounds for providing a unified assessment model for the global network of the Pasteur Institutes. **Method: **This study was designed and performed in 4 stages: first; design of indicators and determining their scores. Second; editing indicators according to the outcome of discussions and debates held with members of Research Council of Pasteur Institute of Iran. Third; implementation of a pilot model based on the Institute’s activities in 2011. Fourth; providing the pilot model feedback to the stakeholders and finalizing the model according to an opinion survey. **Results**: Based on the results obtained, the developed indicators for Pasteur Institute of Iran evaluation were designed in 10 axes and 18 sub-axes, which included 101 major and 58 minor indicators. The axes included governance and leadership, resources and facilities, capacity building, knowledge production and collaborations, reference services, economic value of products and services, participation in industrial exhibitions, status of the institute, satisfaction and institute’s role in health promotion. **Conclusion: **The indicators presented in this article have been prepared based on the balance in the Institute’s four missions, to provide the basis for assessment of the Institute’s activities in consecutive years, and possibility of comparison with other institutes worldwide.

## INTRODUCTION

As one of the principle members of the health and research system, Pasteur Institute of Iran can play an important role in providing the community prevention and control of disease and in achieving the objective of Iran's national future outlook in 2025. Pasteur Institute of Iran is a longstanding member of the International Network of Pasteur Institutes. This network operates with membership of over 30 institutes across the five continents. It is a research, manufacturing, and educational organization that was established in 1919 with the aim of supporting public health and preventing communicable diseases. The necessity for establishing this scientific institution was initially to produce vaccines and to prevent communicable diseases. However, the current policies of this institute include implementing basic and applied research in diagnosis of diseases, providing prevention and control methods, manufacturing biologic and laboratory products, education and research in other medical science fields, holding independent practical units in various specialized areas, and conducting joint educational and research projects with similar centers in national and international levels [1].

An overview of the global network of Pasteur Institutes reveals that, generally, activities of this network members involve in research, public health, education and production, national and international collaborations, interaction with the industry, community etc. [2]. The annual reports of Pasteur Institute in France, with a comprehensive perspective, contain all aspects of research, production, education, clinical etc. [3] for control of both communicable and non-communicable diseases. In Iran, research activities of Pasteur Institute of Iran, much the same as other medical science research centers and institutions, have been evaluated since 2006 [4]. Since this institute is involved in production, services, and educational activities; therefore, it is necessary to design a comprehensive model for assessing all scientific aspects of this institute. Thus, the present study aimed to design a comprehensive performance evaluation model for Pasteur Institute of Iran that can also prepare the necessary grounds for providing a unified assessment model for the global network of Pasteur Institutes.

**Table 1:** Governance and leadership indicators in the evaluation model of Iran Pasteur Institute

**Axis**	**Sub-axis**	**Indicators**
Governance and leadership	Pasteur autonomy	Financial autonomy Autonomy in selecting the president, departmental heads Board of Trustees commensurate with the Institute’s scope of activities
	Strategic program	Program content Program development with the presence of stakeholders, Monitoring and evaluation
	Ethics	Quality and quantity Ethical committee Ethical supervision of research projects

## METHODS

The present study was conducted in 2011 in four stages as follows:

***Stage one. ***Available reports of other Pasteur Institutes as the members of global network of Pasteur Institutes, published reports of the Pasteur Institute in France (as the first in the world), and even the newest Pasteur Institute in China, national comprehensive scientific map and health scientific map in Iran as well as constitutional and strategic plan of Pasteur Institute of Iran were reviewed. Based on the reports from other Pasteur Institutes in the world, generally, activities of most institutes include research, education/training, production, clinical services, and providing professional health services, nutrition, food security, environment, and attention to the necessary infrastructures, such as the physical space, human resources and budget, cooperation with the community and industry, utilization of research findings, the institute’s role in public health, national and international collaborations, being a reference source at national and international levels given the previous history and prospects through outlining the vision, the mission, objectives, and defining strategies.

Through analysis of the mentioned documents, a list of categories and sub-categories of indicators were listed based on the responsibilities, mission, and strategies of Pasteur Institute of Iran and the health research system model [5]. At the next step, a committee consisting of researchers from Pasture Institute of Iran and expert researchers in field of evaluation was established. Over several discussion sessions, a list of objective-oriented evaluation indicators were designed in 10 axes, including “governance and leadership, resources and facilities, capacity building, knowledge production and collaborations, reference services, economic value of products and services, participation in industrial exhibitions, status of the institute, satisfaction and institute’s role in health promotion (consisting of 101 major and 58 minor indicators) (Tables 1- 5).

**Table 2:** Indicators of resources and facilities in Pasteur Institute of Iran`s evaluation model

**Axis**	**Sub-axis**	**Indicators**
Resources	Financial resources	Proportion of Governmental and non-governmental Institute’s funds Pasteur Budget (governmental and non-governmental) as percentage of gross domestic product
	Human resources	Number of Faculty Members, students, and experts Quality of human resources training
	Physical facilities	Quality and quantity of laboratories appropriate for the each department objectives Quantity of educational spaces to the number of students Quality and quantity of consumables, advanced equipments Quality and quantity of production and service units appropriate for strategic program

**Table 3:** Capacity building indicators of the Pasteur Institute of Iran`s performance evaluation model

**Axis **	**Sub-axis**	**Indicators**
Capacity building	Managerial structures in areas of education, research, and services Structure of obtaining support Structure of intellectual ownership of innovation in the indicator Structure of networking and stakeholders` cooperation Product quality structure Structure of knowledge production and technology transfer	Each sub-axis size is determined according to the following criteria: Presence of a developed and approved structure Presence of a responsible person and experienced and competent staff A developed and approved program An up-to-date information web site

***Stage two. ***The developed indicators were discussed with the members of Research Council of Pasteur Institute of Iran, and the model provided was revised according to the members’ comments.

***Stage three. ***According to the values of each indicator and their axes, score of each indicator was determined. Furthermore, a pilot evaluation model was carried out to evaluate the applicability of each indicator in 2011.

***Stage four. ***The results of the pilot study were reported to the Research Council of Pasteur Institute of Iran with the aim of finalizing evaluation indicators and scores, assessing challenges and providing strategies to conduct annual performance evaluation based on the developed indicators. The evaluation model was then finalized.

## RESULTS

The indicators developed for performance evaluation of Pasteur Institute of Iran were designed in 10 axes and 18 sub-axes consisting of 101 major and 58 minor indicators. The axes included: 1) governance and leadership, 2) resources and facilities, 3) capacity building, 4) knowledge production and collaborations, 5) reference services, 6) economic value of products and services, 7) participation in industrial exhibitions, 8) status of the institute, and 9) satisfaction and institute’s role in health promotion. Each axis included a number of sub-axes, each sub-axis had some major indicators, and some major indicators contained one or more minor indicators.

Based on Table 1, governance and leadership of the Institute includes three main components of Pasteur autonomy, strategic program, and ethics. The criteria considered for Pasteur autonomy were autonomy in financial issues and in choosing authorities. In this framework, a Board of Trustees with necessary specialization and skills within the Institute’s scope of activities can make policies in the growing process of financial autonomy and decision making process to select heads of departments.

Other governance criteria included the presence of clear policies and strategies for the Institute’s activities in the form of strategic program, with its indicators considered to consist of content, including having a vision, a mission, and a mission statement, objectives and an action plan, a monitoring and evaluation program, and the ways of other stakeholders participation of in program development.

Finally, a clear ethical program includes ethical committee activity in terms of number of meetings, composition of members based on the approval of the Ministry of Health Research Department, supervision method of the committee on the referral projects in need of assessment and on its implementation process. In total, 159 marks were considered for this axis, which included 130 marks for autonomy of Pasteur Institute of Iran, 19 for strategic program, and 10 for ethics.

According to Table 2, the resources and facilities axis is examined through aspects of financial, human, and physical resources. The sub-axis of financial resources uses the governmental and non-governmental funds absorbed by the Institute, as well as the budget of Pasteur Institute of Iran (governmental and non-governmental) as percentage of gross domestic product to assess the Institute’s ability in attracting funds and maintaining autonomy. The human resource indicators investigates status of the faculty members, students and experts as well as the quality of training of these personnel, whose status in each group in terms of quality and quantity is appropriate for the department/group’s program, number of attracted faculty members, experts and students from brilliant talents or elites, annual rates of students/faculty ratio, and attracting faculty members from outside the institute or abroad as an opportunity or exchange program for professors, Ph.D. students, Ph.D. by research, postdoctoral and fellowship from outside the institute, and the institute’s apprentices at graduate and undergraduate levels. In the indicator of quality of human resources training, the focus is on the number of the institute’s graduates in the evaluation year according to acquired degrees, and number of the Pasteur Institute’s graduates that were recognized as special talents and elites, and also the institute’s graduates’ employment status.

**Table 4:** Indicators of knowledge production and collaborations in Pasteur Institute of Iran`s evaluation model

**Axis **	**Sub-axis **	**Indicators **
4- knowledge production and collaborations	Research projects	According to demand According to type of project
	Publication of articles in scientific/research journals	According to journal indexing According to type of articles Considering Institute foresight in published article According to the author’s rank Publishing joint article with other science organizations
	Citation to the published articles by Pasteur Institute	Mean number of citations Number of citations from Pasteur Institute’s articles in reference books Mean H-index of Pasteur Institute members
	Presentation of articles in congresses	According to congress type According to presentation type
	Publication of books	Writhing scientific books Compilation scientific books
	Holding conferences	National International
	Defended theses	According to educational level (degree) Publication of articles regarding to theses
	Collaboration and communication	Membership in science and technology parks Membership in international clusters Collaboration with the World Health Organization
	Publication of scientific/research journals	According to indexing in data banks (chemical abstract, Scopus, PubMed, ISI, and …)
	Awards	National festivals International festivals
	Inventions, discoveries, and innovations	Inventions in relation to Pasteur Institute’s responsibilities
	Technological and innovation outcomes	Number of incubators Number of knowledge-based companies in terms of subject Number of spin-off companies in terms of subjects
	Providing training and technical advice	According to organization type According to presentation
	Guidelines and summaries	For the public, politicians, service providers, and the industry

**Table 5:** Other indicators in the Pasteur Institute of Iran`s evaluation model

**Axis **	**Sub-axis **	**Indicators **
5- Services reference	Reference for specialized and technology laboratory	National International Regional levels
6- Economic value of products and services	Based on geographic spread of usage	National International
7- Presence in industrial exhibitions	Number of pavilions	National International
8-Institute’s status	National/international	Written public opinions Opinion survey in organizations World ranking among Pasteur Institutes
9- Satisfaction	Satisfaction and organizational loyalty in Institute’s personnel	Faculty members Experts, and other personnel
10- Institute’s role in health promotion	Promotion of equity in health indicators based on stakeholders in Ministry of Health	stakeholders in communicable and non-communicable diseases prevention units

The physical resources were analyzed in terms of the physical space and facilities. These assessment criteria were performed by the opinion of the head of training/research groups in terms of qualitative and quantitative indicators of laboratory commensurate with the groups/department objectives, quality of materials used, advanced equipments and finally quality and quantity of production and service units appropriate for the strategic program.

In total, this axis scored 658 marks consisting of 50 marks for financial resources (the indicator of governmental and non-governmental Pasteur budget as percentage of gross domestic product scored no points), 438 marks for human resources (including 72 marks for the faculty members, 16 for experts, and 350 for students, which according to the nature of quality of human resources training, no limit was considered), and 170 marks for the physical resources.

Based on Table 3, capacity building consists of 7 sub-axes of managerial structures in various areas, obtaining support, institute’s intellectual ownership of innovations and inventions, networking and stakeholders’ cooperation, product quality, knowledge production and technology transfer, and monitoring and evaluation. In addition, according to their criteria, the presence of a developed and approved (by the Board of Trustees) structure, the presence of a trained responsible person with managerial experience and skills, an appropriate developed and approved program, and an up-to-date information web site were determined for each structure. The institute can gain a maximum of 118 marks for capacity building, mostly allocated to the managerial structures in areas of education, research, and services with 60 marks. 

According to the indicators in Table 4, higher marks were allocated to the research projects, based on the request from outside institute (other Pasteur Institutes), other scientific communities abroad, Ministry of Health, other governmental organizations, universities and research centers in the country, and industry in relation to the institute approved research projects. Also, in terms of the project type, higher marks were allocated to the production, intervention, and joint basic and production projects. The indicator of article publication included published articles in scientific/research journals, as the most important criteria for knowledge production in terms of indexing (Pubmed, ISI, Scopus, …), type of article (review, original, case report, and …), the author’s position in writing article rank (first, corresponding, second authors and so on), articles that can envisage the institute’s future (foresight) and joint articles resulting from research projects published by scientific/research organizations and institutions and other universities in the country and abroad. The sub-axis indicators of citation from the published articles by researchers of Pasteur Institute of Iran included mean number of citations of articles with affiliation of Pasteur Institute of Iran, number of citations of articles with affiliation of Pasteur Institute of Iran in reference books, and mean H-index of Pasteur Institute member. The indicator of articles presented in congresses was considered according to congress type (national or international), and in terms of type of presentation (lectures, posters, and invited speakers. Publication of books was investigated in two aspects: writing books based on local research and compilation of the book. Holding conferences was foreseen at national and international levels. Defended theses as well as degree marks were also allocated to published articles considering theses results. The inventions and discoveries were also considered. It should be noted that the criteria for marking the aforementioned indicators of the production and publication of knowledge and collaborations were based on the evaluation of research activities of the country’s universities of medical sciences and research centers. Since 2006, Pasteur Institute of Iran has been evaluated annually according to these indicators [6]

 Other sub-axes and the following indicators in this axis were included in a model for the first time. Thus, no information was available on these sub-axes. The sub-axis of communications and cooperation contained indicators of membership in science and technology parks, membership in international branches/networks, and collaborations with WHO as a “collaborating center”. For the publication of scientific/research journal, based on indexing databanks (Chemical Abstract, Scopus, Pubmed, ISI, and so on), full marks were allocated for first publication, or a percentage was allocated for subsequent years, depending on the type of profile. The indicator of “Awards” achieved in festivals was accounted according to the festival and national or international institutions (various agencies in the United Nations). The sub-axis of technology and innovation, through indicators of number of incubators, number of knowledge-based companies according to subject, number of spin-off companies according to subject, and time of establishment were awarded full marks for the first year, and a fifth of marks for subsequent years.

The sub-axis of training and technical consultations was awarded marks according to type of organization such as other Pasteur Institutes in the world, other scientific/research organizations abroad, the World Health Organization, Ministry of Health and Medical Education, industry and other universities, research centers and scientific institutions of the country, and also according to type of presentation, correspondence, electronic mail, or in person. The sub-axis of regulated guidelines and summaries was considered from the end-user’s perspective (public, politician, service and industry provider) and type of guideline or summary (policy summaries, standards, and clinical guidelines) and means of information transfer (through CD, educational software, or through the media, such as newspapers, radio, and television). In terms of scoring, no limitation was implemented in the collaboration and knowledge production and publication, considering the nature of indicators.

Other indicators are presented in Table 5. All indicators in this Table have been included, for the first time, in the design of an evaluation model for Pasteur Institute of Iran according to studies and review of reports from other Pasteur Institutes, as explained below.

The referential nature of services in terms of laboratory, specialty, and technology at national, international, and regional levels was considered according to the first year or subsequent years of services. The indicators of economic value of products and services and the value of knowledge generated were considered in terms of geographical spread of product and service utilization and marketable production project (inside and outside of the country). The presence in industry exhibitions was assessed in terms of number of pavilions of Pasteur Institute of Iran (in the country and abroad). The status of the institute at national and international levels was considered according to a written opinion of the public visiting the institute, opinion survey conducted in organizations interacting with the institute and other stakeholders. Finally, world ranking of the institute was considered. 

In terms of satisfaction, the level of satisfaction and loyalty to the organization among the personnel of Pasteur Institute of Iran as internal stakeholders (members of faculty and experts) was considered. The most important outlook of the institute, its role in health promotion, will be investigated through its role in promoting equity in health indicators according to the opinion of stakeholders in the Ministry of Health (group discussion with representatives of communicable and non-communicable disease prevention units, representatives of Pasteur Institute of Iran, and the social determinants of health secretariat). All indicators in Table 5 were considered without limitation in scoring according to the nature of indicators. 

## DISCUSSION

Based on the nature of Pasteur Institute of Iran, in the governance and leadership axis, in addition to strategic program indicators and the ethical committee activity considered by Jalalinia *et al.* [7] and considering strategic program as one of the indicators in evaluation model of National Health Research System [8], financial autonomy was also considered, and 80% of marks in the governance and leadership axis were allocated to this sub-axis.

In the resources and facility axis, although Pazargadi *et al.* [9, 10] considered human resources (members of faculty, students, and non-faculty personnel), laboratory and workshop facilities, administrative and financial resources, educational/research space as integral parts of functional indicators in universities of medical sciences and higher education institutes, Jalazadeh *et al.* [11] emphasized on only human and financial resources as an effective input in science and technology growth and development. However in the designed model, in addition to quality and quantity of inputs, their compatibility with strategic plans was also considered. The marks allocated to the indicators in this axis are indicative of its higher importance compared to governance and leadership and capacity building axes, and the most important role in the resource and facility axis belongs to human resources.

In relation to capacity building axis and the presence of structures, only managerial structures in areas of education, research, services, and production were active. Also, creation of other structures based on the discussed indicators will be effective in promotion of the institute in future.

According to the existing evidence, missions of the world’s Pasteur Institutes, including Pasteur Institute of Iran, have been different from those of academic centers. Over the years, the role of Pasteur Institute of Iran in controlling fatal communicable diseases such as plague and rabies and more recently, hemorrhagic fevers support this claim. In recent years, two new responsibilities as education and production have been added to the previous research and service responsibilities of the institute. Of course, in the past, the institute had collaborations with universities and Ministry of Health in terms of professional education and training on the job, and even directly produced B.C.G. vaccines and other products used in prevention and control of communicable diseases. With new missions causing improvement in quality of research and services and eventually community health promotion and equity in health, the national and international credibility of Pasteur Institute of Iran will be enhanced. However, if the attraction of education and production retreats the institute from applied and targeted research and responding to the public health needs, and Pasteur Institute of Iran neglects its holistic and equitable role, undoubtedly, an interminable loss on the country’s healthcare system will ensue. Fortunately, the Board of Trustees of Pasteur Institute of Iran is fully aware of this issue, and has special supervision and attention to the strategic plans and evaluation indicators of Pasteur Institute of Iran. The indicators presented in this article have been produced in accordance with maintaining balance in four missions of the institute, so that, it can provide a basis for evaluation of activities of the institute in consecutive years, and perhaps, for comparison with other institutes in the world. The important point is that the presented indicators and scores are merely suitable for a beginning in evaluation and can be altered in future evaluations. Of course, this should not harm the old mission of the Pasteur Institute of Iran.
